# L-alanyl-L-glutamine ingestion maintains performance during a competitive basketball game

**DOI:** 10.1186/1550-2783-9-4

**Published:** 2012-03-07

**Authors:** Jay R Hoffman, David R Williams, Nadia S Emerson, Mattan W Hoffman, Adam J Wells, Daniele M McVeigh, William P McCormack, Gerald T Mangine, Adam M Gonzalez, Maren S Fragala

**Affiliations:** 1 Sport and Exercise Science, University of Central Florida, 4000 Central Florida Blvd, Orlando, Florida 32816, USA

**Keywords:** Supplement, Dehydration, Hypohydration, Exercise, Reaction Time

## Abstract

**Background:**

The purpose of this study was to examine the efficacy of L-alanyl-L-glutamine (AG) ingestion on basketball performance, including jump power, reaction time, shooting accuracy and fatigue.

**Methods:**

Ten women (21.2 ± 1.6 years; height: 177.8 ± 8.7 cm; body mass: 73.5 ± 8.0 kg), all scholarship NCAA Division I basketball players, volunteered for this study. Subjects participated in four trials, each consisting of a 40-min basketball game with controlled time-outs for rehydration. During the first trial (DHY) subjects were not allowed to rehydrate, and the total weight lost during the contest was used to determine fluid replenishment during the subsequent three trials. During one trial subjects consumed only water (W), while during the other two trials subjects consumed the AG supplement mixed in water using either a low dose (1 g per 500 ml) (AG1) or high dose (2 g per 500 ml) (AG2) concentration. All data assessed prior to and following each game were converted into a Δ score (Post results - Pre results). All performance data were then analyzed using a one-way repeated measures analysis of variance.

**Results:**

During DHY subjects lost 1.72 ± 0.42 kg (2.3%) of their body mass. No differences in fluid intake (1.55 ± 0.43 L) were seen between rehydration trials. A 12.5% (p = 0.016) difference in basketball shooting performance was noted between DHY and AG1 and an 11.1% (p = 0.029) difference was seen between AG1 and W. Visual reaction time was significantly greater following AG1 (p = 0.014) compared to DHY. Differences (p = 0.045) in fatigue, as determined by player loads, were seen only between AG2 and DHY. No differences were seen in peak or mean vertical jump power during any trial.

**Conclusion:**

Rehydration with AG appears to maintain basketball skill performance and visual reaction time to a greater extent than water only.

## Background

Glutamine ingestion during acute dehydration stress is reported to enhance fluid and electrolyte absorption resulting from intestinal disorders [1-3], but it's effects may not be consistent [4]. This is possibly related to stability issues of glutamine in the gut. However, when glutamine is combined with alanine the ability to enhance electrolyte and fluid absorption appears to be greater than glutamine alone, likely via a combination of greater stability and an enhanced rate of absorption via specific ion transporters within intestinal epithelia [5]. In addition, the greater stability of the alanine-glutamine dipeptide appears to be quite evident at a low pH [6]. This could have important implications for athletes during competition.

Recently, acute ingestion of an alanine-glutamine dipeptide (AG) was reported to enhance fluid uptake and reduce the magnitude of performance decrement during exercise to exhaustion under hypohydrated conditions [7]. Furthermore, the alanine-glutamine dipeptide was shown to be significantly more effective than water alone. This has important implications during athletic performance, where dehydration can play a critical role in the outcome of a contest. For instance, a significant performance decrement has been shown with hypohydration levels of only 2% in basketball players [8,9]. This level of hypohydration has been shown to decrease field goal percentage in basketball players by 8%, clearly affecting the potential outcome of a game. Considering that a thirst sensation may not appear until this level of hypohydration has already been reached [10], it becomes critical for athletes to rehydrate even when they do not feel the need to drink. Furthermore, rehydration does appear to be a major issue among basketball players. Nearly half of professional basketball players assessed prior to competitive games were found to be dehydrated prior to the onset of a basketball game, and that fluid intake during the games was not able to compensate for the pregame hypohydration [11]. In light of these findings, it appears that examining rehydration strategies in basketball players is warranted. Thus, the purpose of this study was to examine the efficacy of two different doses (1 g per 500 ml and 2 g per 500 ml) of AG on basketball performance, including jump power, reaction time, shooting ability and fatigue during a basketball game.

## Methods

### Subjects

Ten women volunteered for this study (21.2 ± 1.6 years; height: 177.8 ± 8.7 cm; body mass: 73.5 ± 8.0 kg). Following an explanation of all procedures, risks, and benefits, each subject gave her informed consent to participate in this study. The Institutional Review Board of the University approved the research protocol. Subjects were not permitted to use any additional nutritional supplementation during the course of the study. Screening for additional supplement use was accomplished via a health history questionnaire completed during subject recruitment. All subjects were scholarship athletes playing for the University's Women's basketball team. The study protocol was a double-blind cross-over design.

### Testing protocol

Data collection occurred on four separate occasions. Each session required subjects to participate in a 40-min basketball game (normal duration for a NCAA college basketball game). To simulate an actual competition, a 2-min time out was used at the 10-min mark of each half, and a 10-min halftime separated the first and second halves. Subjects were divided into two equally talented teams as determined by the team's player captains. The team members remained the same for each game. Thus level of competition (subjects competing against each other) was the same for each contest. Interestingly, each team won two games. The difference between each contest was the type of hydration fluid that was provided. During the first session (DHY) subjects were not allowed to rehydrate. During this session the total weight lost during the contest was determined, which was then used to determine the fluid replenishment during the subsequent three experimental sessions. During these three sessions subjects were provided fluid every 10 min in equal amounts for a total of six hydration times. The fluid consumed at each ingestion point was equal to the fluid loss observed during session one, divided by six. During one of these sessions subjects consumed only water (W), while during the other two session subjects consumed the AG supplement marketed as Sustamine™ (Kyowa Hakko USA, New York, NY) mixed in water using either a low dose (1 g per 500 ml) (AG1) or high dose (2 g per 500 ml) (AG2) concentration. The order of the three sessions was randomly determined per subject. All subjects were expected to begin each game in a euhydrated state. Prior to each contest a urine sample was analyzed for urine specific gravity (U_sg_) by refractometry to document euhydration; U_sg _≤ 1.020 was defined as euhydration [12]. If a subject's U_sg _> 1.020 she was requested to ingest 500 ml of water and retested.

### Performance measurements

Prior to each testing session all subjects performed a 10-min dynamic warm-up. This warm-up was the same warm-up these athletes performed prior to every game during the competitive season. Following the warm-up subjects performed power, reaction and basketball shooting assessments. All testing sessions were supervised by certified strength and conditioning specialists. At the conclusion of the basketball game and final hydration intake, subjects performed all performance measures. Order of performance testing was performed in a randomized fashion for both pre-game and post-game assessments. Test-retest reliabilities for all assessments were R > 0.90.

### Power

To quantify vertical jump power subjects performed five consecutive countermovement jumps (CMJ). During each jump subjects stood with their hands on their waist at all times. The subjects were instructed to maximize the height of each jump while minimizing the contact time with the ground between jumps. During each jump the subject wore a belt connected to a Tendo™ Power Output Unit (Tendo Sports Machines, Trencin, Slovak Republic). The Tendo™ unit consists of a transducer attached to the end of the belt which measured linear displacement and time. Subsequently, the velocity of each jump was calculated and power determined. The average peak and mean power outputs for all five jumps were recorded.

### Reaction

**Figure 1:**
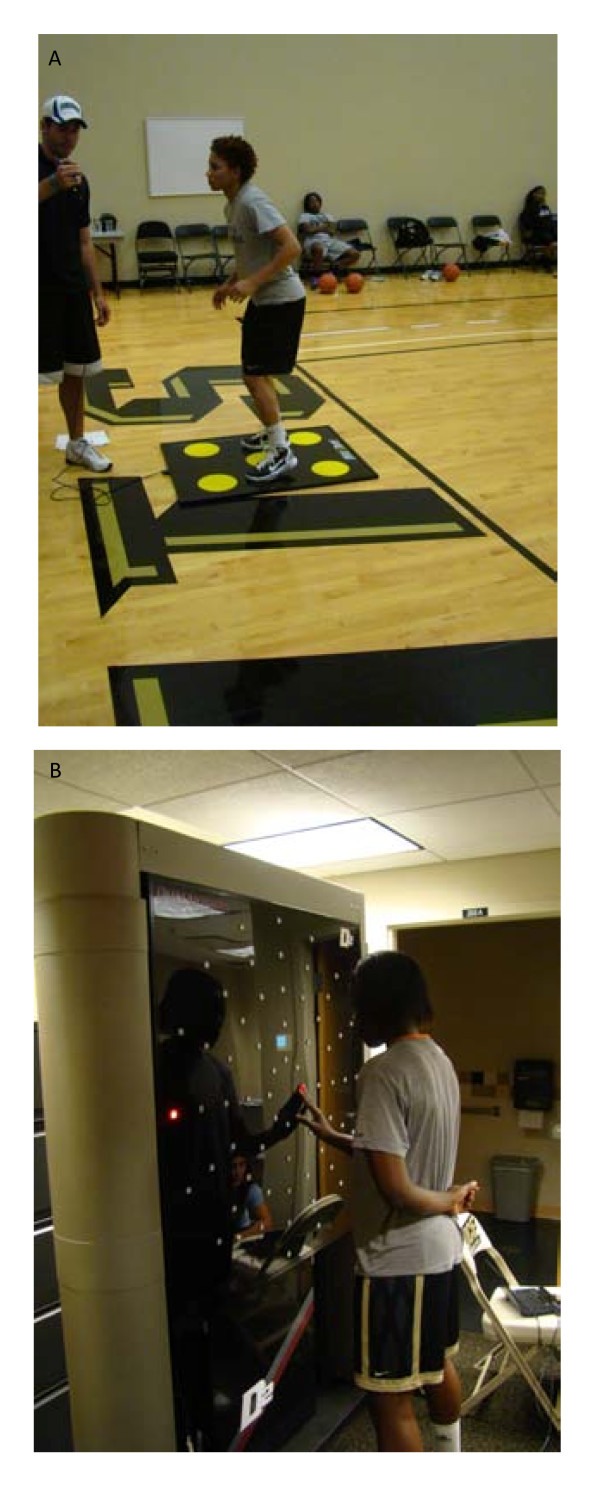
**a) Quick Board; B) Dynavision D2**.

Lower body reaction time was measured with a 20-s reaction test on the Quick Board™ (The Quick Board, LLC, Memphis, TN) reaction timer. Subjects stood on a board of five circles, in a 2 × 1 × 2 pattern (see Figure 1a). Subjects straddled the middle circle and reacted to a visual stimulus located on a display box that depicted one of five potential lights that corresponded with the circles on the board. Upon activation of the light the subject attempted to move the foot closest to the circle that corresponded to the visual stimulus. Upon a successful connection the next stimulus would appear. The total number of successful attempts for the 20-s test was recorded.

Measurement of hand-eye reaction time was performed on the Dynavision D2 (Dynavision, Ontario Canada). Subjects were required to assume a comfortable athletic stance and stand at a distance from the board where they could easily reach all of the lights (see Figure 1b). The board height was adjusted so the LCD screen was located just below eye level. Participants were told to fixate their gaze on the LCD screen in the middle of the board and to keep their focus there for the entirety of the experiment. During the assessment each subject pressed a light with their dominant side index finger on the board. When a second light flashed (on the same line of the initial light, but on the non-dominant side of her body), the subject removed her finger and pressed the new visual stimulus. The time necessary to recognize the new stimulus (new light lit until finger lifted from initial light) was recorded as visual reaction time, and the time it took for the subject to move and press the newly lit light was recorded as the motor reaction time. The total time for both visual reaction and motor reaction was calculated as the physical reaction time. A total of eight attempts were performed. The average time for all eight attempts was recorded.

### Player load and heart rate

All subjects were provided with an individual global positioning system (GPS) that they wore in a vest underneath their playing jersey. The GPS unit (MinimaxX, V4.3, Catapult Innovations, Victoria, Australia) was positioned in a posterior pocket on the vest situated between the subject's right and left scapula in the upper-thoracic spine region. Since the subjects were playing in an indoor facility, there was no viable connection to satellite technology prohibiting information on velocity and distance of activity. However, the ability to measure all gravitation forces (G force) in the G_Z_, G_X_, G_Y _planes of movement were present. The G forces accumulated during the course of each contest were defined as the Player Load. Player load is an accumulated rate of change of acceleration calculated with the following formula:

$$\text{Player Load}=\overset{\text{t = n}}{\underset{\text{t = 0}}{\sum }}\sqrt{\left({\left(\text{fw}{\text{d}}_{\text{t = i + 1}}-\text{fw}{\text{d}}_{\text{t = i}}\right)}^{2}+{\left(\text{sid}{\text{e}}_{\text{t = i + 1}}-\text{sid}{\text{e}}_{\text{t = i}}\right)}^{2}+{\left(\text{u}{\text{p}}_{\text{t = i + 1}}-\text{u}{\text{p}}_{\text{t = i}}\right)}^{2}\right)}$$

Where: Fwd = forward acceleration; side = sideways acceleration; up = upwards acceleration; i = present time; t = time.

Data was collected at 10 Hz and analysis was performed with the system software provided by the manufacturer. The validity and reliability of GPS technology has been demonstrated in several studies [13,14], and specific validity of accelerometry and player load in evaluating basketball performance has also been reported [15].

Heart rates were continuously monitored with the Polar FT1 (Polar Electro, Kempele, Finland). Each subject placed the heart rate strap underneath their sports bra. All heart rate data was captured by the GPS unit and downloaded to the GPS computer system following each experimental session.

### Basketball shooting performance

**Figure 2:**
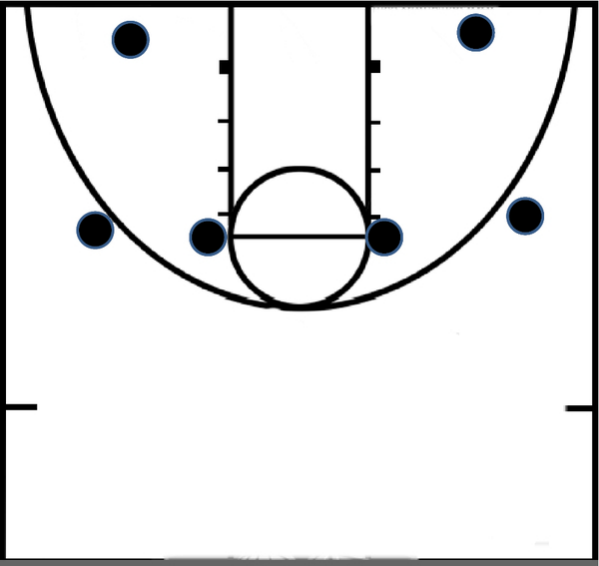
**Basketball Shooting Performance**.

Prior to, and following each game a pre-determined basketball shooting circuit was performed. The circuit required all subjects to shoot 5 balls from 6 different locations on the court (see Figure 2). The total number of successful shots was recorded. The difference between the pregame and post-game shooting performance was calculated and analyzed.

### Sweat rate determination, fluid ingestion, and body mass measures

During the experimental session in which no water was provided subjects were weighed pre and post game. The difference in body mass was attributed to sweat loss. The total body mass loss was used to determine fluid intake in the subsequent experimental sessions. The total fluid loss was recorded and then divided by six. That amount of fluid was provided to each subject at regular intervals. Initial hydration occurred prior to the onset of the game, at minute 10 of the first half, at minute 20 of the first half, prior to the onset of the second half, 10 min of the second half, and at minute 20 of the second half (at the games conclusion). Subjects were instructed to consume the fluid provided, but were not required to drink the entire amount if they did not feel comfortable. Total water consumed by all subjects was recorded. Body mass was determined 10 min prior to the warm-up and immediately following post-game data collection.

### Statistical analysis

Since the primary purpose of this investigation was to examine the efficacy of different hydration strategies on the ability to maintain basketball performance, all data assessed prior to and following each game were converted into a Δ score (Post results - Pre results). All performance data were then analyzed using a one-way repeated measures analysis of variance. In the event of a significant F-ratio, *post hoc *comparisons using the Fisher's least square difference method was applied to determine pairwise differences. A criterion alpha level of p ≤ 0.05 was used to determine statistical significance.

## Results

**Figure 3:**
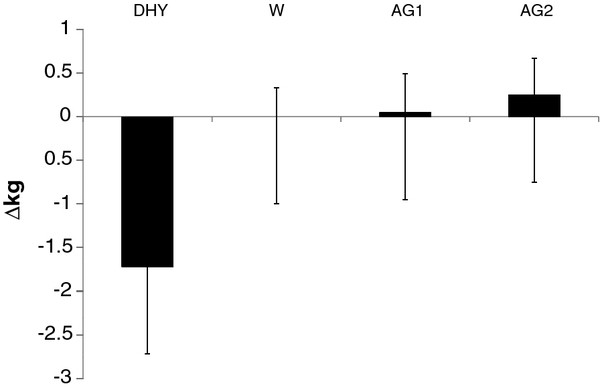
**Change in Body Mass**. * = significantly different (*p *< 0.05) than W, AG1 and AG2. All data are presented mean ± SD.

The temperature and relative humidity for all games were consistent (22.6 ± 0.19°C, and 50.9 ± 3.1%, respectively). All subjects began each game in a euhydrated state (U_SG _= 1.018 ± .008). No significant differences (*p *= 0.472) in U_SG _were seen between trials. During DHY subjects lost 1.72 ± 0.42 kg, this was equivalent to a 2.3% loss of their body mass. This was significantly greater than that seen during any other experimental trial (Figure 3). Fluid intake was not significantly different between W, AG1 and AG2 (1.55 ± 0.43 L).

**Figure 4:**
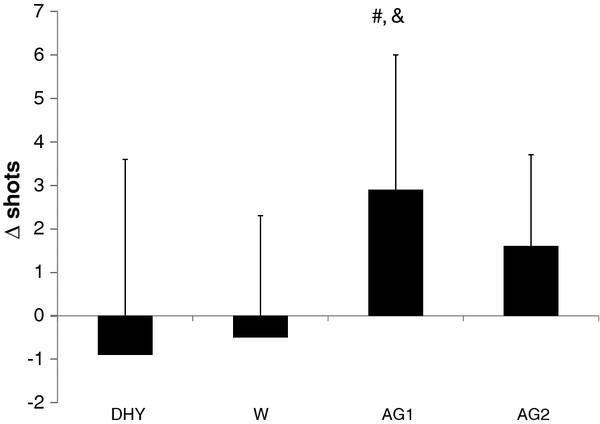
**Field Goal Shooting**. # = significantly different than DHY; & = significantly different than W. All data are presented mean ± SD.

A significant difference was noted between DHY and AG1 (*p *= 0.016) in the controlled shooting drill (see Figure 4), and a trend was seen between AG2 and DHY (*p *= 0.094). Furthermore, shooting performance was significantly better between AG1 and W (*p *= 0.029). During the AG1 trial subject's shooting percentages were 12.5% and 11.1% greater than DHY and W, respectively.

**Figure 5:**
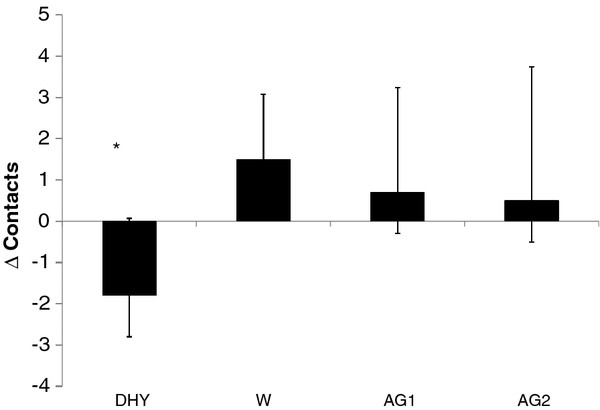
**Change in Lower Body Reaction**. * = significantly different (*p *< 0.05) than W, AG1 and AG2. All data are presented mean ± SD.

**Figure 6:**
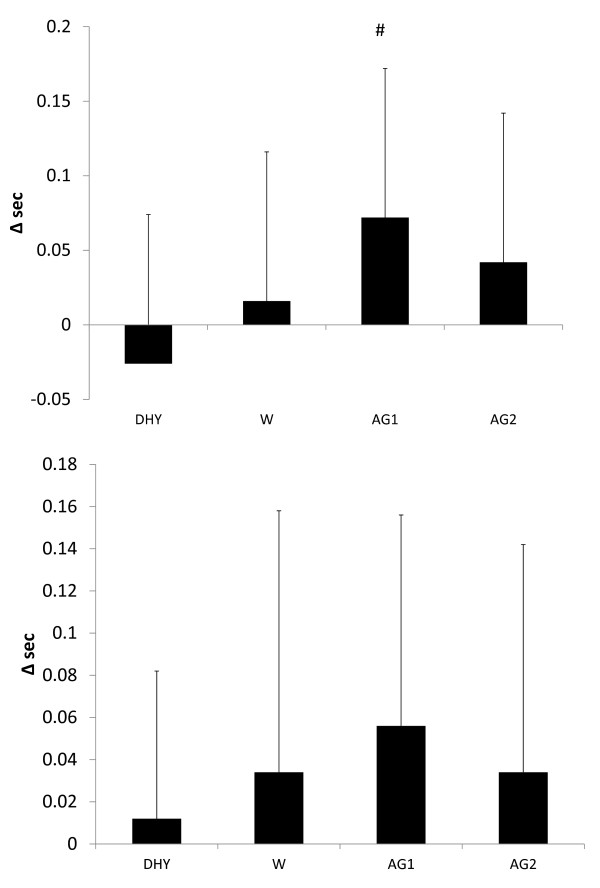
**Change in a: Visual reaction time**. # = significantly different than DHY; b: Motor reaction time. All data are presented mean ± SD.

A significant difference in lower body reaction was seen between DHY and the other experimental trials (see Figure 5). No further differences between trials were noted. Visual reaction time (Figure 6a) was significantly better following AG1 (*p *= 0.014) compared to DHY, and a trend toward a similar response (*p *= 0.081) was noted between AG2 and DHY. However no significant differences were noted in the motor response (see Figure 6b). The change in the physical reaction time (combined visual and motor differences) was significantly greater for AG1 compared to DHY (*p *= 0.032).

**Figure 7:**
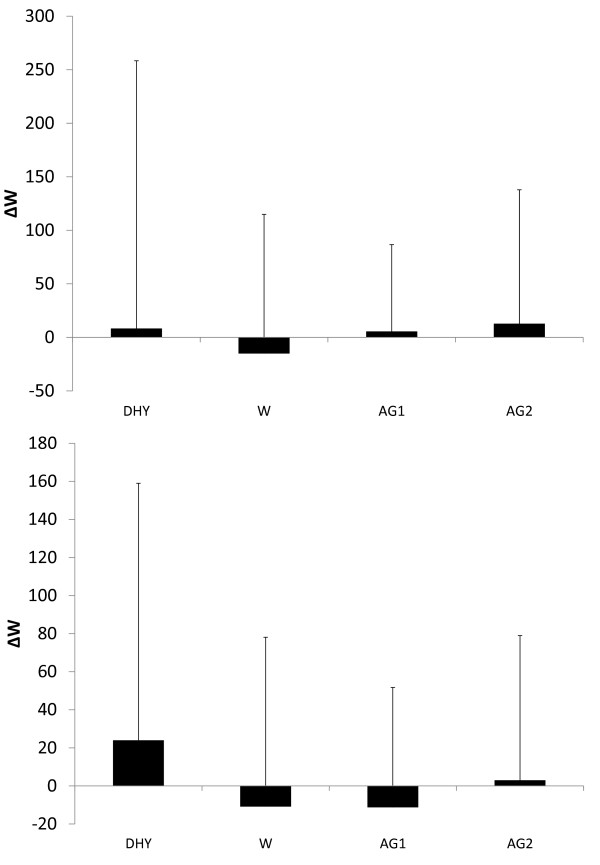
**Change in: a = Peak Vertical Jump Power; b = Mean Vertical Jump Power**. All data are presented mean ± SD.

**Figure 8:**
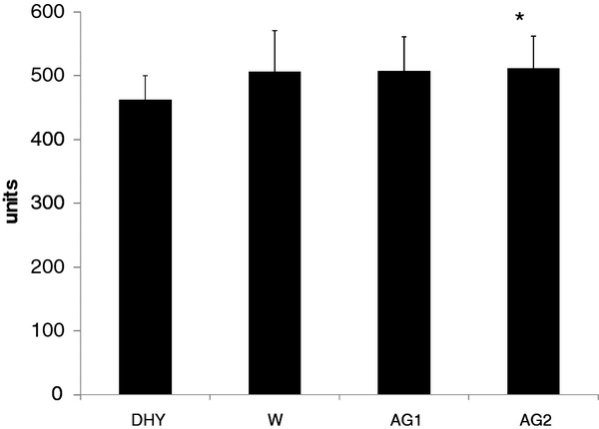
**Player Load**. # = significantly different than DHY. All data are presented mean ± SD.

No significant differences were seen in the pre to post game differences in either peak or mean vertical jump power (see Figures 7a and 7b, respectively). Figure 8 depicts the player loads calculated from the GPS device during each game. During AG2 a significantly greater player load was seen compared to DHY (*p *= 0.045). A trend for greater player loads were also noted between AG1 (*p *= 0.064) and W (0.073) compared to DHY. Average heart rates during each experimental trial are depicted in Table 1. No significant differences were noted in average heart rate between each trial. Although heart rates were 4.5% to 5.3% lower in all trials compared to DHY, these differences were not statistically different.

**Table 1:** Average Heart Rates

	First Half	Second Half	Entire Game
**DHY**	176.8 ± 8.2	174.5 ± 7.5	175.7 ± 7.3

**W**	169.2 ± 9.9	164.6 ± 15.9	166.8 ± 10.8

**AG1**	167.7 ± 13.4	168.5 ± 9.7	168.1 ± 11.2

**AG2**	166.9 ± 11.9	166.5 ± 13.3	166.7 ± 12.3

**P value**	0.186	0.286	0.200

All data are presented as mean ± SD

## Discussion

Results of this study indicate that female basketball players lose approximately 2.3% of their body mass during a game in which they are not permitted to rehydrate. Despite a significant loss of body fluid during DHY subjects were able to maintain jump power throughout the game, but basketball shooting performance and reaction time was significantly impaired. Rehydration trials using AG was able to maintain basketball shooting accuracy to a better extent than water alone, and ingestion of AG1 also enhanced visual reaction time. Subjects consuming the supplement were able to respond to a visual stimulus quicker than when dehydrated. No significant differences in visual reaction time were observed in subjects ingesting water compared to the dehydrated condition. Lower body reaction time was significantly reduced when subjects were not permitted to rehydrate, however no differences were seen between water and AG ingestion.

The level of hypohydration seen in this study was similar (2.3% versus 2.0%) to previous research examining a 40-min basketball game in men [9]. The effect of this mild hypohydration stress on jump power performance was consistent with previous research examining the effect of mild to moderate levels of hypohydration on jump or repetitive jump performance [9,16,17]. Judelson and colleagues [17] showed that jump power is maintained following dehydration protocols that elicited a 2.5% and a 5.0% loss of body mass. Similarly, Cheuvront et al., [16] also reported no decrement in jump power performance in men following a 3.8% loss in body mass. Taken together, it appears that mild to moderate levels of dehydration do not impair jumping performance

Dehydration has been shown to impair basketball shooting performance in several studies [8,9,18]. While Hoffman and colleagues [9] reported that a 2% level of dehydration can decrease shooting percentages by 8% (results not statistically different), others have shown that a similar level of hypohydration can cause significant performance decrements in shooting accuracy [18] and that it can progressively decline with greater levels of fluid loss [8]. The results of this present investigation are consistent with these latter studies. The mechanism that may have contributed to a decrease in shooting percentage may be fatigue relating to the hydration stress. However, considering that power outputs remained consistent between experimental trials and no difference in player load was observed between DHY and AG1, it is more likely that other factors contributed to the differences observed in shooting percentages between DHY and AG trials.

A recent investigation has indicated that moderate levels of dehydration (4% body mass loss) can result in significant alterations in afferent neural processing [19]. This suggests that the ability to maintain fine motor control in performance, such as shooting a basketball, may become significantly impaired during a hypohydration stress. Additional research has also indicated that dehydration can increase lateral ventricle enlargement in the brain causing a higher level of neuronal activity in the brain required to achieve the same performance level [20,21]. This may explain in part the significant performance decrements observed in reaction time (both visual and in lower body) during DHY. When subjects were permitted to rehydrate (regardless of W or AG) lower body reaction times were significantly improved. However, the ingestion of AG1 significantly enhanced basketball shooting performance to a greater extent (*p *< 0.05) than W only. In addition, AG1 improved visual reaction time during the competition, whereas no difference was observed between W and DHY. Although not statistically different, similar trends were seen between AG2 and shooting accuracy and visual reaction time (*p *= 0.09 and *p *= 0.08, respectively). The ability to enhance visual reaction time with AG1 does appear to have important implication for athletic performance. Mann and colleagues [22] have suggested the ability to process visual information provides critical information for enhancing the anticipatory response during athletic performance. Achieving excellence in basketball has been suggested to be related in part to an ability of the athlete to have a "highly-tuned" anticipatory ability that allows them to predict other's actions ahead of their realization [23].

Rehydrating with AG during the rest breaks of the game may have contributed to a more efficient fluid and electrolyte uptake, minimizing the deleterious effects of dehydration. Previous research has demonstrated that the AG dipeptide can enhance fluid and electrolyte absorption in the gut, especially at a low pH, which is common during exercise [5,6], and that when consumed by subjects during a mild hydration stress (-2.5% of body mass loss) exercise can be prolonged to a greater extent than with water ingestion only [7]. Although speculative, AG ingestion may have augmented fluid uptake from the gut, and minimized the potential deleterious effects that mild levels of dehydration had on nerve conduction and brain function. These effects may be more prevalent in activities involving multisensory information such as shooting (involves a coordinated and precise visual and motor control of the hands and arms) versus reaction of the lower body.

In conclusion, rehydration with AG appears to maintain basketball skill performance and visual reaction time to a greater extent than water only. These effects are likely mediated by enhanced fluid and electrolyte uptake from the gut and subsequent preservation of neural function that commands physical activities involving fine motor control. Further research appears warranted in the examination of AG ingestion and neural activity during periods of hydration stress.

## Competing interests

The authors declare that they have no competing interests.

## Authors' contributions

JRH was the primary investigator, designed study, supervised all study recruitment, data/specimen analysis, statistical analysis and manuscript preparation. DRW, NSE, MWH, AJW, DMN, WPM, GTM and AMG were co-authors, assisting with data collection and data analysis. MSF helped drafting the drafting the manuscript. All authors read and approved the final manuscript.
